# Alginate Hydrogel Formulation with Ketorolac for the Treatment of Pain Related Sialolithiasis

**DOI:** 10.3390/gels9050415

**Published:** 2023-05-16

**Authors:** Cristina Silva, Gladys Ramos-Yacasi, Mireia Mallandrich, Helena Colom-Codina, Joaquim Suñer-Carbó, Noelia Pérez-González, Ana Cristina Calpena, Francisco Fernández-Campos

**Affiliations:** 1 Departament de Farmàcia i Tecnologia Farmacèutica, i Fisicoquímica, Facultat de Farmàcia i Ciències de l’Alimentació, Universitat de Barcelona (UB), 08028 Barcelona, Spain; 2 Facultad de Ciencias Farmacéuticas, Bioquímicas y Biotecnológicas, Universidad Católica de Santa María (UCSM), Arequipa 04001, Peru; 3 Institut de Nanociència i Nanotecnologia (IN2UB), Universitat de Barcelona (UB), 08028 Barcelona, Spain; 4 Department of Pharmacy and Pharmaceutical Technology, Faculty of Pharmacy, University of Granada, 18071 Granada, Spain

**Keywords:** ketorolac, gel, hydrogels, alginate, sialolithiasis, mucosa, inflammation

## Abstract

Sialolithiasis mainly affects the oral salivary glands due to the presence of small stones that obstruct the secretion of saliva. The treatment and control of pain and inflammation during the course of this pathology is essential to guarantee the patient’s comfort. For this reason, a ketorolac calcium cross-linked alginate hydrogel was developed, and it was then applied in the area of the buccal cavity. The formulation was characterized (swelling and degradation profile, extrusion, extensibility, surface morphology, viscosity, and drug release). The drug release was studied ex vivo in static Franz cells and with a dynamic ex vivo method under artificial saliva continuous flow. The product exhibits adequate physicochemical properties considering the intended purpose, and the drug concentrations retained in the mucosa were high enough to deliver a therapeutic local concentration able to reduce the pain associated with the patient’s conditions. The results confirmed the suitability of the formulation for application in the mouth.

## 1. Introduction

Sialolithiasis is considered one of the most common salivary gland disorders in the lives of young adults, though sometimes it can also arise in teenagers and among elderly patients [1,2,3].

**Figure 1:**
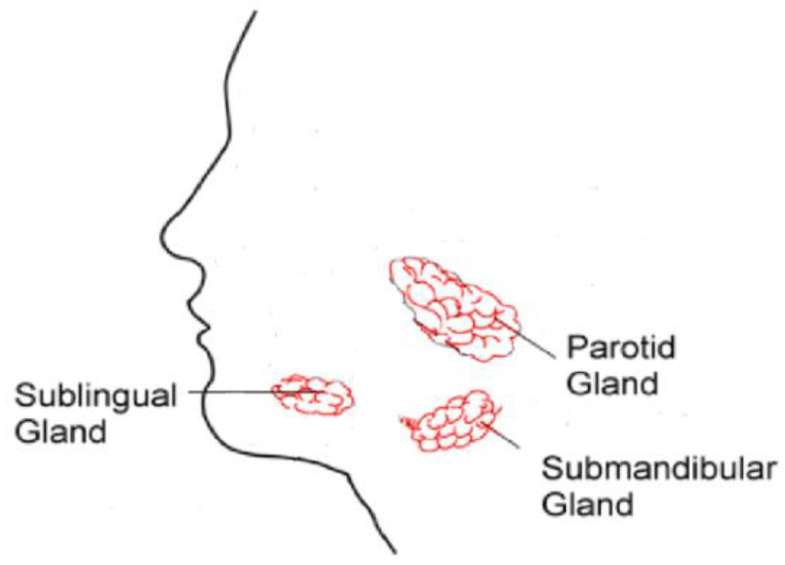
Layout of the salivary glands.

The sialolithiasis pathogeny is represented by a mechanical obstruction of the salivary glands (Figure 1) or its excretory duct by the presence of a salivary stone. It is formed by deposits of calcium, phosphate, and magnesium salts and can be single or multiple, mobile or impacted, and their shape can differ, being oval or rounded [4]. The obstruction to regular salivary flow causes intraductal pressure, which leads to periodic attacks of pain and an acute swelling in the area involved [2].

Since the 1990s, the treatment of sialolithiasis has undergone substantial changes to obtain a minimally invasive healing process and to avoid total or partial resection of the salivary gland [5]. The nature of the salivary stones, such as composition, consistency, size, patient aspects, and the gland involved, defines the surgical or non-surgical management of sialolithiasis to be used. Pharmacological therapy usually includes the use of spasmolytics, antibiotics, and anti-inflammatory drugs.

The non-surgical option includes conservative treatments, which involve physical therapy, massages, sialogogues, and drinking plenty of fluids to facilitate salivary stone expulsion front the duct. In complex cases, extracorporeal shock-wave lithotripsy, methods of transoral duct surgery, and interventional sialendoscopy could be employed as physical treatments (5). However, in both pathways, pain, trismus, and inflammation are treated in the post-medical intervention so as to recover salivary flow.

Oral anti-inflammatory drugs are the most commonly selected form in order to improve patient comfort. Likewise, the purpose of developing novel drug delivery devices is focused on preventing drug side effects and reducing dosing intervals, thus local delivery systems represent good pharmaceutical ways to achieve this [6]. In this context, the use of topical hydrogels is an attractive option as a buccal mucoadhesive dosage forms due to their minimal systemic toxicity and their having sufficient penetrating properties to be effective. In these systems, the drug is uniformly dispersed in the polymer matrix, and drug release is controlled by diffusion through the polymer network. They are composed of high amounts of water and polymer, which increase the formulation mechanical strength as well as residence time in the buccal cavity [7,8].

Alginate is an anionic copolymer composed of 1,4-β-D-mannuronic acid and α-L-guluronic acid, obtained from brown sea algae (Phaeophyceae) [9]. The most commonly available salt is sodium alginate. Alginate is one of the most widely used natural polymers in the drug delivery field and biomedical applications. It is an economical, readily available, biocompatible, biodegradable, and non-toxic polysaccharide that offers countless possibilities in the pharmaceutical and food industry [10].

According to Park and Robinson studies, polyanion polymers present more bioadhesive potential than polycation polymers or nonionic polymers [11]. Thus, alginate as a polyanion polymer with carboxyl end groups presents excellent mucoadhesive attributes. These attributes were evaluated using different polymers (alginate, polystyrene, chitosan, carboxymethylcellulose, and poly(lactic acid)) and ex-vivo intestinal epithelium. It was reported that alginate had the highest mucoadhesive strength among the catalogue of tested polymers [12]. An additional consideration is that its aqueous solubility and affinity for divalent ions, such as calcium, make it a promising polymer for various controlled release applications [13].

**Figure 2:**
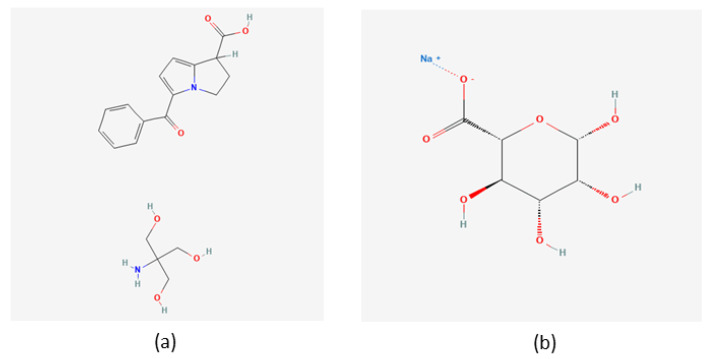
Chemical structures of: (**a**) ketorolac tromethamine [17] and (**b**) sodium alginate [18].

Ketorolac, a hydrophilic non-steroidal anti-inflammatory drug that inhibits cyclooxygenase (COX) 1 and 2 enzymes (Figure 2), has been dispensed as oral tablets or by intra-muscular injection for buccal treatments. Ketorolac is recommended to control pain, trismus, and swelling after lower third molar extraction even if the surgical intervention is highly difficult (10 mg four times daily by sublingual route), and it can be administered by intra-oral injection or the intra-canal for endodontic pain [14,15,16].

In this work, cross-linked alginate hydrogels loaded with ketorolac were prepared so that they could be used in the treatment of inflammatory buccal conditions such as sialolithiasis. This hydrogel can play an important role in the prevention of pain and inflammation as part of a strategy that, among other benefits, would alleviate the symptoms during a preliminary period, until the medical intervention day takes place. Additionally, it would aid patient comfort as a classic postoperative medication. Likewise, it could be used during non-surgical treatments to achieve salivary stone expulsion.

The hydrogels were characterized by rheological analysis, extensibility, and scanning electron microscopy. Texture and physical stability were chosen as the first criteria for ketorolac’s formulation selection. Mucoadhesiveness, syringeability, and swelling tests characterized the appropriate physicochemical properties of the gel, and in vitro release and ex vivo permeation studies were developed so as to prove they had sufficient penetrating properties to be effective as topical analgesics.

As far as we know, this is the first study into the relationship between alginate hydrogel formulation-loaded ketorolac and pain reduction in the oral mucosa. The aims of the current study were to develop cross-linked alginate hydrogel loaded with ketorolac for the treatment of inflammatory buccal conditions such as sialolithiasis, which facilitated the therapeutic compliance of the patient, and thus assessed the suitability of the hydrogel for the buccal delivery and enables investigation into its biocompatibility with the buccal and sublingual mucosae [19].

## 2. Results and Discussion

### 2.1. Formulation of the Alginate Gel with Ketorolac

An initial screening was carried out to select the formulation with the physicochemical properties best suited for the intended use. The initial formulations created by using 50 and 100 mM CaCl_2_ did not result in the expected gel (it was too thick for it to be administrated) and, therefore, its concentration was decreased. Table 1 shows the gelation properties, physical stability, and visual viscosity after one week of production.

G14, G15, and G16 were initially selected, of which the G15 gel formulation was chosen due to the texture it presented when placed on the skin. G14 was considered too liquid to adhere to the mucosa and the G16 was too thick for it to be easily administered with a syringe. Therefore, the gel composition selected contains 2% alginate, 10 mM calcium, and 1% ketorolac. Furthermore, the pH value is within the physiological pH range of the mouth cavity.

### 2.2. Physicochemical Characterization of Kerotolac Hydrogel

#### 2.2.1. Appearance Evaluation and Sensory Properties

The appearance of a hydrogel is key to find out the desired consistency and texture according to the required application because inadequate sensory properties of a formulation can lead to poor adherence to the therapy resulting in therapy failure [20]. A visual evaluation of the stability allowed the evaluation of the sensory hydrogel’s properties and characteristics over a long period of time. It is very important that the hydrogel has the same appearance as the hydrogel without phase separation and that it can be demonstrated that this pharmaceutical form is stable.

Appearance is the first factor to be taken into account in terms of the stability of a pharmaceutical form. This will allow the development of a suitable formula that retains the same physicochemical properties for patient acceptability during a selected period. A large number of studies dealing with the stability of hydrogels are available [21].

**Figure 3:**
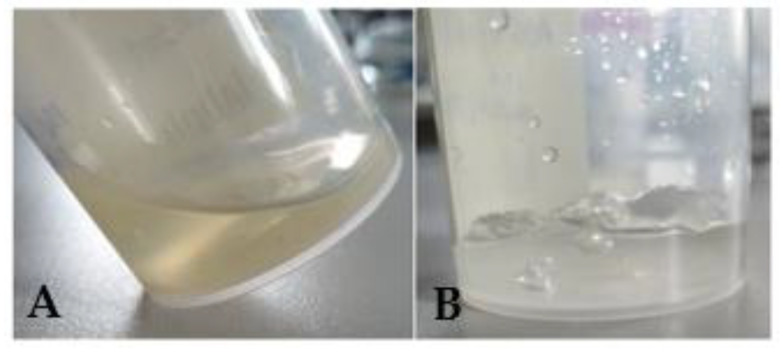
Appearance of KT alginate gels before reticulation (without the crosslinking agent CaCl_2_) (**A**), and G15 formulation, KT alginate gel cross-linked with the addition of CaCl_2_ (**B**).

Figure 3 shows the two steps to make the gel: first, there was a ketorolac alginate gel to which there was added CaCl_2_ as a cross-linker (in other words, before reticulation) and the gel was reticulated (G15) with the incorporation of a crosslinking agent (CaCl_2_). Once the calcium had been added, the alginate gel’s viscosity increased, resulting in a limited flowability (Figure 3B) in contrast to the gel before adding CaCl_2_, which flowed when tilting the flask (Figure 3A). The non-reticulated gel was yellowish-translucent, odorless, and slightly sticky to the touch, whereas the reticulated gel (G15) was viscous and transparent.

The G15 formulation was evaluated over 6 months stored at room temperature conditions, there being no macroscopic changes in appearance and sensorial properties. In addition, the formulation underwent further characterization (Fourier transform infrared, extensibility, morphological, evaluation of the porosity, swelling and degradation behavior, mucoadhesivity, extrudability, rheologically, biopharmaceutically, and histologically). Results are reported in the following sections.

#### 2.2.2. FT-IR

**Figure 4:**
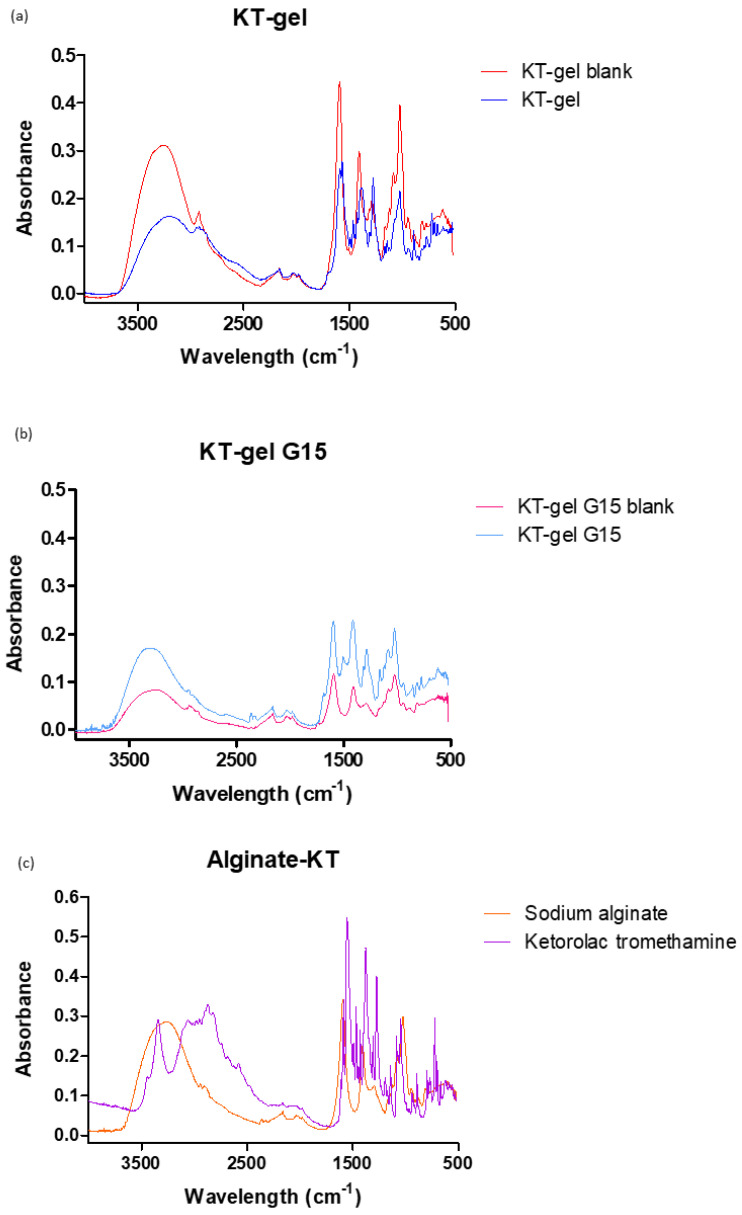
FT-IR spectra for alginate gels: (**a**) red = dried placebo of KT alginate gel (prior reticulation) and blue = dried of KT alginate gel (prior reticulation); (**b**) red = dried placebo of KT G15 gel and blue = dried KT G15 gel; and (**c**) red = sodium alginate powder and blue = ketorolac powder.

To investigate whether any interaction occurred between the drug and the polymer, the hydrogel was studied by Fourier transform infrared (FT-IR). Figure 4 shows the FT-IR spectra for KT G15, KT G15 placebo, and the gel before adding the cross-linking agent and its placebo, as well as the spectra for alginate and KT substances.

All spectra showed strong peaks around 1600 cm^−1^ (COO^−^ stretching) present in both alginate and ketorolac. No bands disappeared nor did new bands appear, indicating that no interactions between KT and alginate occurred.

#### 2.2.3. Extensibility

**Figure 5:**
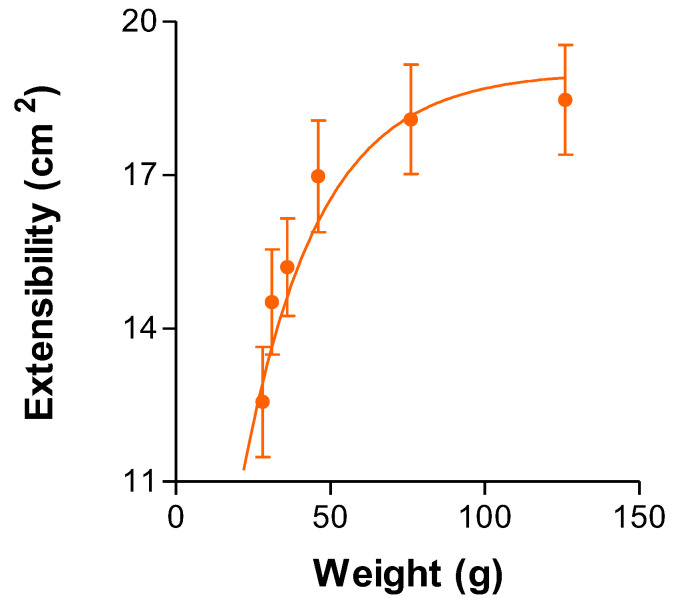
Extensibility of the formulation G15 depending on the weight applied fitting first-order kinetic model.

The extensibility of the gel indicates how the gel spreads from the time of application. The extensibility of the G15 formulation, in its equilibrium swelling state, can be seen in Figure 5. It shows that the more weight applied over the top plate, the greater the extensibility obtained.

The extensibility model follows a first-order kinetics, whose parameters are: Y_max_ = 19.02 cm^2^ representing the maximum value of extensibility in the test, K = 0.041 g^−1^ being the extensibility constant and HL = 17.07 g being the half-life. In this way, it spreads quickly over a large surface with little weight, reaching its maximum extensibility; even if more weight is applied the extensibility is not increased. It reached a maximum of 19.02 cm^2^ when applying 126.06g. With every 17g of hydrogel applied, the extensibility increased by half. These results imply less force and pressure in the gel application process in sensitive or painful areas due to the pathology of sialolithiasis. As the application of a drug, it must be as simple and painless as possible.

The texture and extensibility of the hydrogel make its application in the affected area easier [22].

#### 2.2.4. Morphological Studies

SEM was used to evaluate the morphology of the hydrogel’s internal structure with ketorolac. Previous research [23] showed that the internal structure of alginate (4% *w*/*w*) hydrogel without calcium reticulation exhibits a needle-like structure. The fiber structures we obtained are more pointed and ordered compared with the non-reticulated hydrogel, showing the effect of the calcium crosslink.

**Figure 6:**
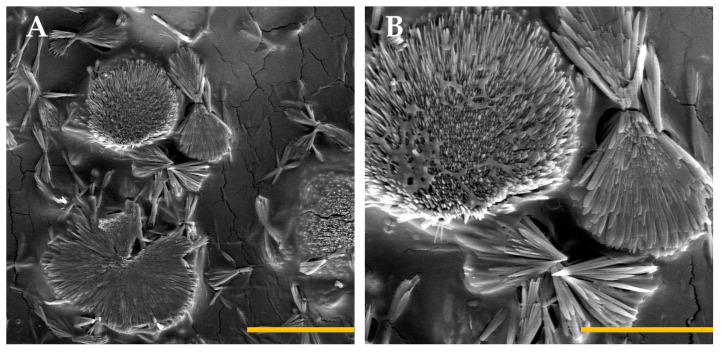
Scanning electron microscopy images of KT hydrogel G15 at different magnifications: 2000× and scale bar of 50 µm (**A**) and 5000× and scale bar of 20 µm (**B**). The alginate and crosslinking agent (CaCl_2_) were mixed in a 1:1 for the final formulation.

The KT hydrogel G15 images obtained when the hydrogel is reticulated with calcium ions showed a flower-shaped conformation in which the filaments start from a common area and open. Between conformations, there are spaces to store water and the dissolved ketorolac. The fiber structures obtained are more pointed and ordered compared with the non-reticulated hydrogel, showing the effect of the calcium crosslink (Figure 6).

#### 2.2.5. Determination of the Gel Porosity

Hydrogels are composed of polymers that form three-dimensional structures or networks. The arrangement of the polymer chain will determine the mesh size and shape, which in turn will determine the gel porosity [24]. Porosity refers to the fraction of void volume related to the total volume [24]. The porosity of G15 KT hydrogel was calculated by immersing the dried gel in ethanol and evaluating the solvent uptake according to Equation (2). The porosity resulted in 28.4 ± 1.8%. The porosity of gels may have an impact on the swelling, drug release profiles, and other characteristics [25]. For instance, Nindiyasari and co-workers investigated the influence of the porosity of a gelatin-based hydrogel on the growth of CaCO_3_ crystals; the researchers observed that higher amounts of gelatin in the hydrogel resulted in smaller crystals [26]. Choi et al. were able to tune the porosity of hydrogels loading mRNA [27].

#### 2.2.6. Swelling and Degradation Tests

**Figure 7:**
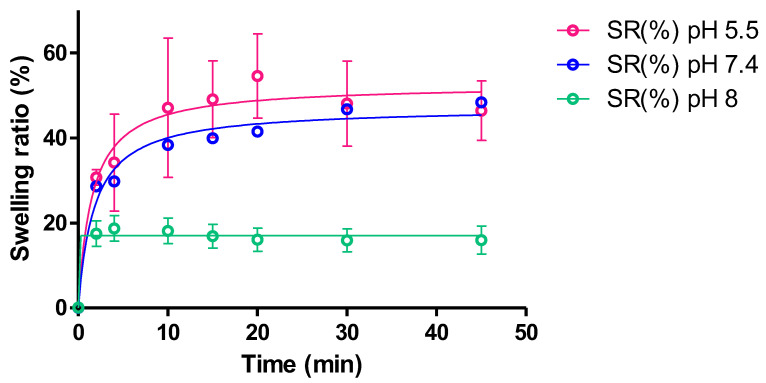
Swelling ratio of dried KT hydrogel G15 upon immersion in PBS at the different pH of the buccal cavity (*n* = 3 for each pH). The swelling ratio followed the hyperbola model.

Swelling experiments were carried out to evaluate the ability of the dried hydrogel to incorporate solvent, because the mesh size of the hydrogel may impact the swelling ratio affecting the solvent uptake, which dissolves the drug in the matrix and, hence, modulates the drug release into the tissue. The swelling was evaluated at three pH values. Figure 7 shows the kinetics model and the obtained swelling parameters. As can be observed, the swelling followed a hyperbola model, going up to a maximum fold increase of about 50 times the initial weight of the hydrogel for pH 5.5 and 7.4; a remarkable decrease in the swelling rate was observed (17.02) at pH 8. The dry polymer exhibits a high hygroscopicity. The quick solvent uptake could also be the result of the high porosity of the formulation (observed in SEM images) and the hydrophilic behavior of the polymer fibers. Other authors reported similar results for reticulated alginate performance. Da Silva et al., 2009 evaluated the swelling degree of alginate film at 5% and 10% of polysaccharide, and the film swelled 0.12 and 0.15 times its initial weight in about 10 min [28]. Moussaoui et al., 2021 evaluated the non-cross-linked alginate hydrogel at 4% (*w*/*w*), and this lead to the conclusion that it increased its weight 20-fold in 10 min [23]. Moussaoui et al. elaborated an alginate gel (4% *w*/*w*) enriched with hyaluronic acid (5% *w*/*w*), which increased the initial weight 15-fold when immersed in PBS [29].

Table 2 shows the results of the mathematical modelling of the swelling data. 

**Figure 8:**
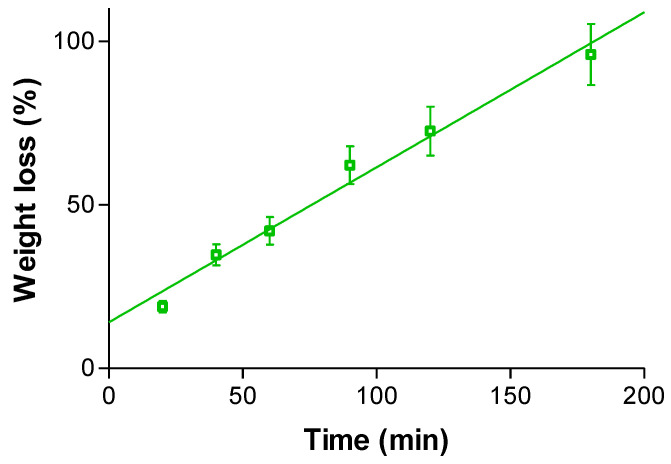
Degradation of G15 KT hydrogel in PBS pH 7.4, which fitted a zero-order kinetic model. Each value represents the mean ± SD (*n* = 3).

Figure 8 shows the degradation behavior of KT hydrogel G15 when it was immersed in PBS pH 7.4. A constant degradation (zero-order kinetics) of 0.474% per minute was obtained, and the hydrogel was completely disintegrated after 180 min. In a real situation, after disintegration, the polymer would be swallowed and go to the gastrointestinal tract, where alginate would behave as dietary fiber. These experimental conditions did not completely mimic the in vivo conditions, because the formulation would not be completely soaked in liquid under in vivo conditions, but the saliva would be in touch only with the formulation surface. To reduce the degradation, it would be recommended to apply the hydrogel at night, when the saliva flow and the mouth movements are lower. In that way, the contact time with the oral mucosa would also be higher. 

#### 2.2.7. Ex Vivo Mucoadhesion Study

**Figure 9:**
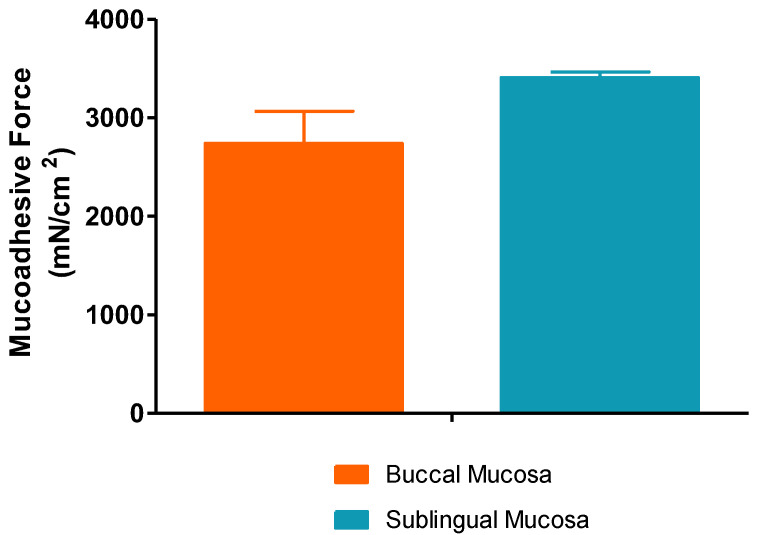
Mucoadhesive force (mN/cm^2^) of hydrogel developed with porcine buccal mucosa and sublingual. Each value represents the mean ± SD (*n* = 3).

The adhesiveness is a very important factor for a formulation applied to the oral cavity because it determines the contact time with the target site and the diffusion of the drug to the underlying tissue. Mucoadhesive force values with buccal and sublingual porcine mucosa are shown in Figure 9. There were statistical differences in the mucoadhesion on both tissues (2739.6485 ± 325.7437 and 3407.9390 ± 57.0992 mN/cm^2^ for buccal mucosa and sublingual, respectively). This fact is probably due to the sublingual mucosa being less keratinized than the buccal. These differences in mucoadhesion could be related to some structural aspect of the tissue. As seen in the histological analysis (Section 2.6), greater cell differentiation and/or a more superficial membrane thickness can be observed on the surface of the epithelium, which may be related to the membrane-lining granules [30]. This fact may be associated with the production or content of sialic acid, which is responsible for the mucoadhesion with the polymer. The results obtained confirmed the good mucoadhesive properties of the hydrogel and its suitability for oral application.

#### 2.2.8. Syringeability/Extrudability Study

Studies have been conducted to show that syringeability is important in determining the maximum force and work required to expel formulations from the syringe in medication application [31].

Depending on the disease location on the buccal cavity, it could be difficult to obtain a high drug bioavailability. The syringe administration device is an attractive option to obtain precise contact on these areas. One of the important aspects of the characterization is to measure the maximum force and work required to expel formulations from the syringe in medication application [31] and so to evaluate the ease of removing the formulation from the container and to apply the product.

**Figure 10:**
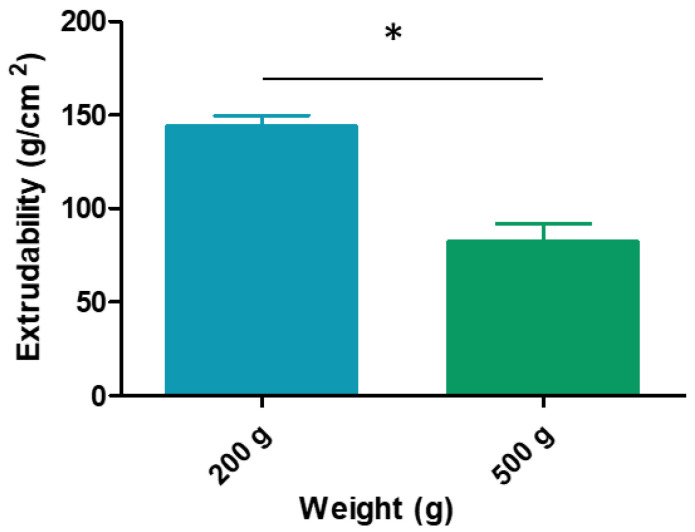
Extrudability (g/cm^2^) behavior of hydrogel developed measured with a 2 mL syringe. Each value represents the mean ± SD (*n* = 3). * significant statistical difference *p* < 0.05.

To evaluate the convenience for the patients and the ease of administration with a syringe, the syringeability evaluation was carried out with 2 mL syringes. The syringeability measurement is based on the extrusion capacity of the formulation to be released from the device after the application of different pressures. Results are reported in Figure 10. The extrudability values were 143.3761 ± 6.3247 when a weight of 200 g was applied and 81.8570 ± 10.1607 when a weight of 500 g was used. According to these results, the hydrogel loaded in the syringe shows good extrusion properties, which means the patient does not have to apply a great force to administer it.

#### 2.2.9. Rheological Analysis

**Figure 11:**
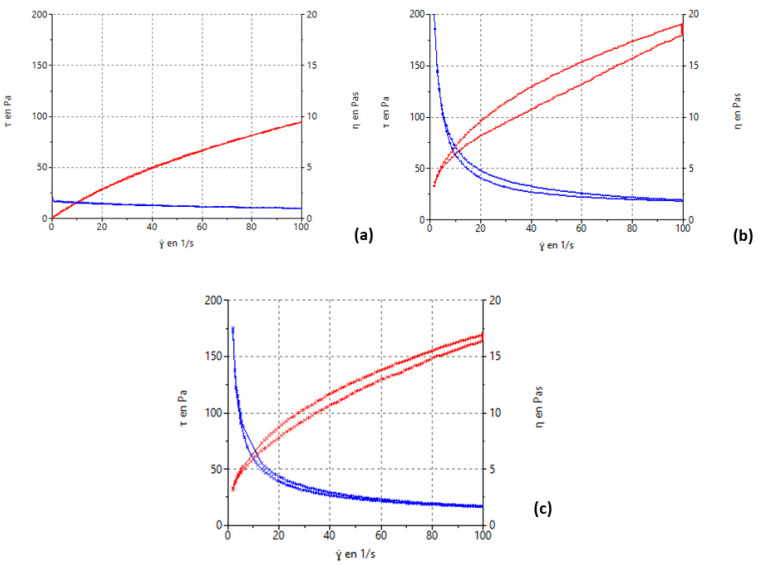
Rheological behaviour of G15 KT hydrogel: (**a**) before being reticulated, (**b**) reticulated gel, and (**c**) extruded reticulated gel. Viscosity curve (blue line, right axis, η in Pa·s) and flow curve (red line, left axis, τ in Pa) of G15 Kt hydrogel as a function of shear rate (γ in s^−1^).

The rheological profile of semisolid formulations is important for their appropriate characterization because it determines the stability and the sensorial properties, as well as the behavior in storage and during administration. Steady-state rheological measurements of the selected formulation (G15) as a function of shear rate are shown in Figure 11. Formulations exhibited non-Newtonian pseudoplastic flow and shear-thinning behaviour with a decrease in viscosity with increasing shear rate from 0 to 100/s^−1^. The shear stress versus shear rate values were fitted to the Cross equation (Table 3) as to confirm the rheological behavior. 

The pseudoplastic profile is interesting for the intended use of the formulation. At rest, the viscosity of pseudoplastic material is higher than the viscosity when a shear stress is applied, for example when the product is rubbed on the body surface or when the product is extruded by the syringe. This improves stability of the product at rest and eases the administration by the patient. The pseudoplastic behavior is confirmed by the fit to the Cross equation. These results agreed with the results obtained with other alginate hydrogels [23].

### 2.3. In Vitro Release Study

**Figure 12:**
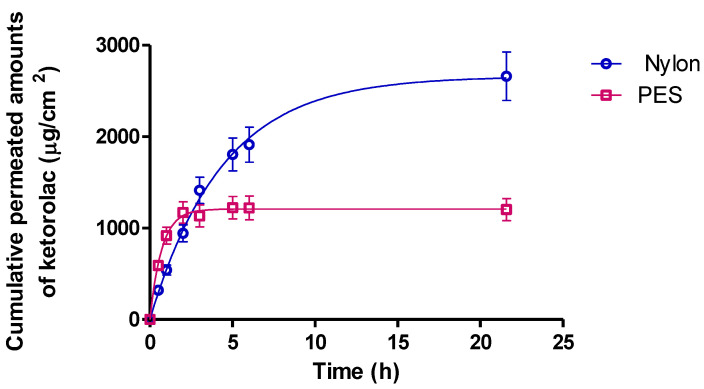
Cumulative amount of ketorolac released from the hydrogel in artificial membranes, PES, and nylon.

In vitro drug release experiments were performed on Franz-type cells to evaluate the ability of the hydrogel formulation to release the drug. Two different synthetic membranes were evaluated to select the membrane that gives less resistance to the drug diffusion. This fact allows the evaluation of the release mechanism, which should not be limited by the membrane. As can be seen in Figure 12, polyester sulfone (PES) limited the drug release, probably due to the more hydrophobic nature compared to nylon, and considering that KT is highly hydrophilic, PES is not considered the best membrane with which to study the drug released.

Once the membrane (nylon) was selected, the data set was fitted to different kinetic equations: hyperbolic, first-order, and Higuchi. The model was selected based on the higher determination coefficient (R^2^), which corresponds to a first-order equation (Table 4). Then, the drug release followed Fick’s law, according to the release being governed by diffusion, as a function of the remaining drug concentration in the formulation.

The maximum release was obtained after 22 h of the study with a value of 2666 µg/cm^2^, which corresponds to 85.14% of the drug applied. The first-order kinetic parameters are reported in Table 5.

Moussaoui et al., 2021 [23] reported similar results with a 2% KT gel in 4% alginate, obtaining similar results in both the maximum amount released and the release constant. Despite having half the polymer compared with Moussaoui, being cross-linked with calcium slows down the release of the drug, obtaining equivalent results having a double concentration of the polymer.

### 2.4. Ex Vivo Permeation through Buccal and Sublingual Mucosa

To evaluate the absorption of KT through the buccal mucosa and the sublingual mucosa, ex vivo permeation experiments were performed in line with different methodologies previously reported [32,33,34], in both cases under an infinite-dose regimen [35].

**Figure 13:**
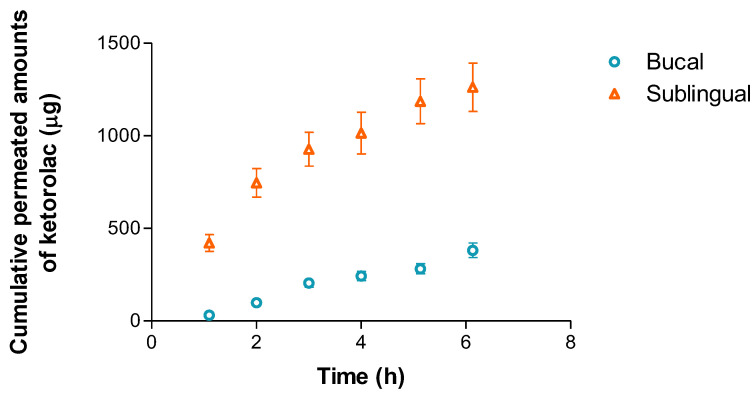
Cumulative amount of ketorolac permeated from the hydrogel through buccal and sublingual mucosae. Results are represented as the mean ± SD of six replicates.

The permeation profiles of the KT through the buccal and sublingual mucosae are shown in Figure 13, and the cumulative amount of KT permeated through the oral mucosae shown in the parameters calculated can be seen in Table 6 [36,37,38].

All the parameters evaluated were higher after the product application in the sublingual mucosa, compared to buccal mucosa. This fact is probably due to the membrane not being as thick [39], also shown in Figure 15. The good permeability profile of KT was previously reported in other studies. El Moussaoui et al. [23] evaluated the skin and vaginal KT permeation dose at 2% (*w*/*v*) in no-reticulated alginate hydrogel (4% *w*/*v*). The sublingual flux was higher than the vaginal flux as well as the Kp value. As expected, the mucosal permeation was much higher than the reported skin values due to the lack of stratum corneum.

To evaluate if the permeated concentrations would produce significative plasma levels, the concentration at steady state (Css) was calculated based on two possible surfaces, 1 cm^2^ and 2 cm^2^. The therapeutic plasma concentrations were previously reported by Cordero et al. [40] being 0.3–5 µg/mL. To obtain a local effect, and not a systemic effect, the application surface on the sublingual mucosa should be 1 cm^2^. It is worth remembering that the present study was carried out under an infinite dose scheme (150 mg formulation/cm^2^) that is much higher than the dose that would be used in clinical conditions (usually between 2–15 mg formulation/cm^2^ [41]). These infinite conditions are usually carried out to maximize the permeation and to evaluate the formulation properties and the permeation mechanism. It could also be considered as a safety screening experiment in cases of overdose. Even if the prescribed dose was 150mg/cm^2^, the Css levels would not be above the maximum plasma concentration value, and then the proposed formulation can be considered safe.

### 2.5. Evaluation of the Drug Amount Released and Retained in the Buccal/Sublingual Mucosa under an Artificial Saliva Flow

The mucosa permeation experiment (Section 2.4) was carried out in static conditions, with no saliva flow. To conduct a more realistic in vivo approach, a dynamic experiment was performed. A constant saliva flow was simulated with a peristaltic pump that flowed on the excised pig buccal mucosa with the formulation on the surface. After the experiment, the KT retained in the mucosa tissue was extracted and analyzed by HPLC.

**Figure 14:**
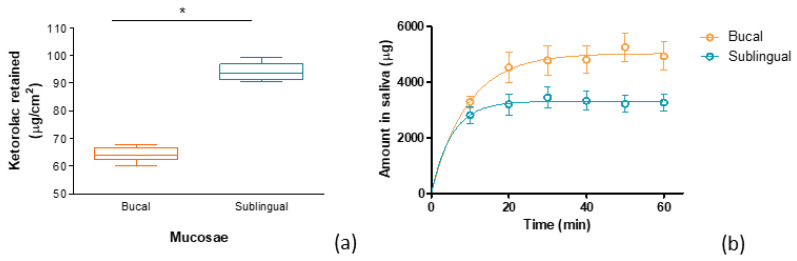
(**a**) Amount of ketorolac extracted from the buccal and sublingual mucosa after ex vivo dynamic study with a constant flow of artificial saliva, and (**b**) amount of KT dragged by the artificial saliva flow during the ex vivo dynamic study. * Statistical significant differences (*p* < 0.05).

As can be seen in Figure 14, the KT content in sublingual mucosa was significatively higher compared to the buccal mucosa, but in both cases, significant KT concentrations were found. The higher content in sublingual mucosa is probably due to it being thinner, and this accelerates the drug absorption [30]. This experiment corroborates the good permeation profile shown in the previous experiment (Section 2.4). The KT content in the target site (mucosal tissue) is correlated with the analgesic and anti-inflammatory effects of the drug.

The amounts of KT found in the saliva were about 1.5-fold higher for the buccal mucosa than the sublingual mucosa, and probably because of this fact, the amount of KT found in the buccal mucosa was lower than that found in the sublingual mucosa. The different behavior of saliva on dragging KT from one mucosa or another may be related to the adhesive capacity of the formulation to the buccal or sublingual mucosa (Figure 8). Taking into account that the mucoadhesivity of KT G15 hydrogel was greater for the sublingual mucosa, it suggests that the hydrogel remains in contact longer with the sublingual mucosa, and this fact reduces the clearance of the formulation by the saliva resulting in a higher drug availability in the sublingual mucosa.

### 2.6. Mucosa Histology Evaluation

**Figure 15:**
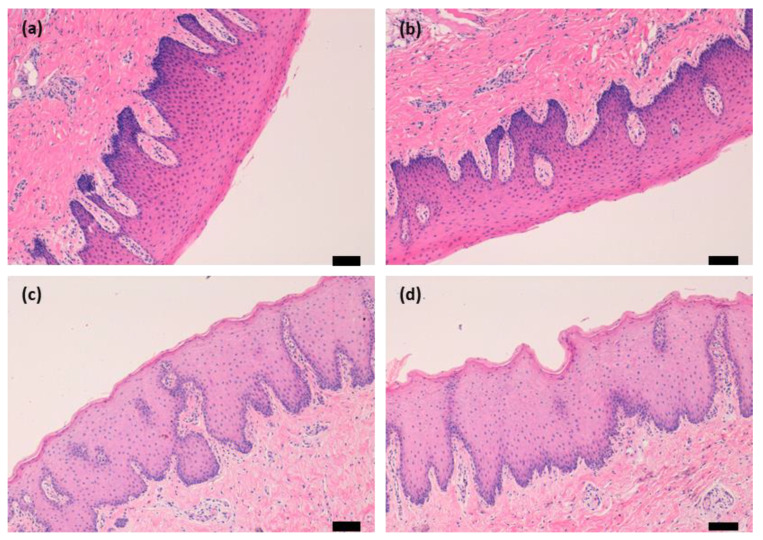
Histological sections of mucosae after the ex vivo permeation study: (**a**) Sublingual mucosa histology under basal conditions, without gel (100× magnification); (**b**) sublingual mucosa histology after gel exposure (100× magnification). (**c**) Buccal mucosa histology under basal conditions, without gel (100× magnification); (**d**) buccal mucosa histology after gel exposure (100× magnification). Scale bars = 100 µm.

To evaluate the mucosa integrity used on permeation experiments, a histological study was carried out. Tissue (buccal and sublingual mucosa) samples were taken just after the animal extraction, which corresponds to the viable tissue. Tissue samples used after the permeation experiments (Section 2.4) were also taken. Both were hematoxylin-eosin-stained and examined under a light microscope (Figure 15). As can be seen in the figure, both mucosae (buccal and sublingual) presented a normal stratified epithelium with no significant structural alterations.

## 3. Conclusions

A hydrogel based on alginate (2% *w*/*w*) cross-linked with calcium (10 mM) ketorolac (1% *w*/*w*) as an active ingredient was developed and characterized. The formulation had adequate rheology characteristics, extrudability, extensibility, and physical properties to be administrated on the buccal cavity with a syringe. The drug was released from the formulation quickly, high extended, and effectively delivered into the tissue, with a high mucoadhesive profile to stick to the tissue.

The permeation of ketorolac into the tissue was good under the static infinite dose approach and under constant saliva flow, simulating in vivo application. To obtain a local effect, it is recommended that the formulation is applied in the buccal mucosa on 1 cm^2^ surface.

The results show that the formulation of KT hydrogel is a suitable and stable formulation for the treatment of inflammatory processes in the buccal cavity, such as sialolithiasis or dental pathologies.

## 4. Materials and Methods

### 4.1. Material

Sodium alginate was purchased from Fagron Iberica (Barcelona, Spain). Calcium chloride (CaCl_2_), ketorolac tromethamine (KT), and nipagin were purchased from Sigma-Aldrich (Barcelona, Spain). Nipasol from Acofarma (Barcelona, Spain) and Na_2_HPO_4_, KH_2_PO_4_, and NaCl by Panreac (Barcelona, Spain). Methanol from Merck (Darmstadt, Germany). The purified water was obtained from a Milli-Q1 Gradient A10 system apparatus (Millipore Iberica S.A.U. Madrid, Spain). All the other chemicals and reagents used in the study were of analytical grade.

### 4.2. Tissues and Experimental Animals for Ex Vivo Assays

Buccal and sublingual pig mucosa (Landrace Large White race) was provided by the Bellvitge animal facility service. The Ethics Committee of Animal Experimentation of the University of Barcelona approved the Study Protocol (approved on 10 January 2019). The mucosa was cut to 500 µm with mucotomed (Aesculap^®^, B.Braun, Barcelona, Spain).

### 4.3. Formulation of the Alginate Gel with Ketorolac

Solutions of alginate diluted in water with preservatives (methyl and propyl paraben, 0.2 and 0.02% *w*/*w*, respectively) were created in different percentages, 1%, 2%, and 4% *w*/*w*. Ketorolac (2% *w*/*w*) was added to the polymer solution and stirred until complete dissolution before cross-linking. At the same time, calcium chloride solutions with concentrations of 8, 16, 20, 24, 50, and 100 mM were also prepared.

The alginate and calcium chloride solutions were mixed in a 1:1 volume ratio and allowed to stand for 24 h to evaluate cross-linking. After dilution, the final concentration of ketorolac was 1% in all the formulations. Table 7 shows the different formulations prepared and their composition.

After 24 h post-production, the formulations were studied in terms of appearance. Hydrogel texture and signs of instability (precipitation and other macroscopic alterations) were considered for a preliminary screening so as to select the formulation for further characterization.

### 4.4. Physicochemical Characterization of Kerotolac Hydrogel

#### 4.4.1. FT-IR

The hydrogels and their components were investigated for chemical interaction between KT and the polymer by Fourier transform infrared. The samples investigated were: KT alginate gel prior to reticulation, KT G15 gel, both placebos, and alginate and ketorolac powders. All the KT gels and placebos were dried in an oven at 55 °C. FTIR spectra were obtained using a Nicolet iZ10 spectrometer (Thermo Scientific, Waltham, MA, USA). The measurements were performed in the range of 4000–525 cm^−1^ with a DTGS detector using a spectral resolution of 4 cm^−1^, obtaining 32 scans per spectrum. The spectra were recorded using attenuated total reflectance (ATR) with a diamond crystal.

#### 4.4.2. Extensibility

One gram of the selected formulation at the equilibrium swelling state was placed between two methacrylate plates. Different weights (2 g, 5 g, 10 g, 20 g, 50 g, 100 g) were then placed on the upper plate, and the formulation surfaces were recorded. The extensibility was obtained with Equation (1).

(1) $$\mathrm{Extensibility}=\frac{\pi \;d^{2}}{4}$$

where *d* is the mean of the diameter of the measured shafts. The data were fitted to a first-order model.

#### 4.4.3. Morphological Studies

To study the microstructure of the hydrogel by scanning electron microscopy (SEM), the sample must be desiccated and covered with carbon or metal when they are non-conductive samples.

For this, a small amount of KT hydrogel G15 was desiccated in an oven at 55 °C and mounted on SEM holders once dried. Then, the sample was coated with a thin film of gold and observed by a QUANTA FEI 200 FEG-ESEM system in the Institute of Materials Science of Barcelona (ICMAB-CSIC), Universitat Autònoma de Barcelona.

#### 4.4.4. Determination of the Gel Porosity

The porosity of the gel was determined by immersing the dried hydrogel in ethanol. The gel was dried in an oven at 55 °C until a constant weight of the gel was achieved. A weighed amount of dried hydrogel was immersed in ethanol and kept at 37 °C. The increase in weight of the hydrogel was monitored for 27 min (until no increase was observed) and the porosity was calculated based on Equation (2):

(2) $$P=\frac{W_{s}-W_{d}}{\rho \times V_{s}}\;$$

where *W_d_* is the weight of the dried hydrogel, *W_s_* is the weight of the swollen hydrogel, ρ is the density of ethanol, and *V_s_* is the volume of the swollen hydrogel determined by a pycnometer.

#### 4.4.5. Swelling and Degradation Tests

A gravimetric method [32] was used to evaluate the uptake of phosphate buffer solution (PBS) determining the swelling ratio (*SR*). The freshly prepared formulation (25 mg) was deposited on the surface of a glass container and placed in the oven at 55 °C until the weight was constant and the dry gel was obtained. Small pieces of dried hydrogel were immersed in 0.5 mL PBS at three different pH values within the range of the oral cavity pH (5.5–8). Three replicates for each pH were included in the study; the pieces of desiccated hydrogel were placed in Eppendorf at 37 °C for 45 min and at the following time points 2, 4, 10, 15, 20, 30, and 45 min; the Eppendorf were centrifuged at 3000× *g* r.p.m. for 3 min; then the supernatant PBS was pipetted and the Eppendorf was weighed to observe the solvent uptake by the gel. Next, 0.5 mL of fresh PBS were added to the Eppendorf, which was placed back in the thermostatic water bath until the next time point. The *SR* was obtained according to Equation (3):

(3) $$SR=\frac{W_{s}-W_{d}}{W_{d}}\;$$

where *W_d_* is the weight of dried hydrogel and *W_s_* is the weight of the swollen hydrogel at different times.

The degradation test of the hydrogel was done by recording the weight loss as a time function. Weight loss (*WL*) was calculated by incubating about 8 g of fresh hydrogel in PBS pH = 7.4 (the physiological pH of the oral cavity) at 37 °C for 3 h. The hydrogel was extracted at 20, 40, 60, 90, 120, and 180 min, and the excess of water was blotted and weighed at the different times in triplicate. It was calculated based on Equation (4):

(4) $$WL\left(\%\right)=\frac{W_{i}-W_{d}}{W_{i}}100\%\;$$

where *W_i_* is the initial weigh of hydrogel and *W_d_* the weight of hydrogel at different times.

#### 4.4.6. Ex Vivo Mucoadhesion Study

**Figure 16:**
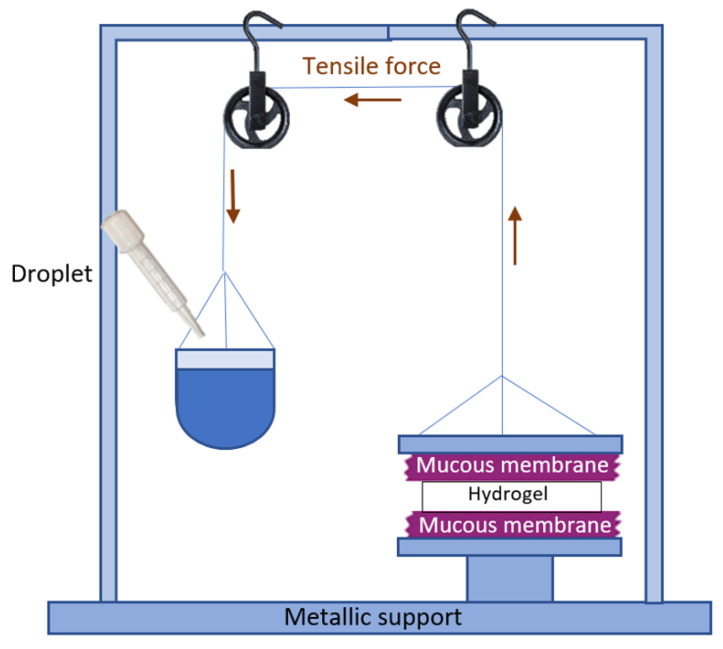
Simplified representation of the device to determine ex vivo mucoadhesion.

The ex vivo mucoadhesive properties are based on the measurement of the resistance or tension offered by the sample when trying to break the bond between the tissue epithelial membrane and the formulation. For this purpose, and based on other devices designed by other authors [42,43,44,45], a simple device was developed to determine the mucoadhesive force of the formulation; Figure 16 depicts a schematic representation of the instrument used, and mucoadhesion was evaluated as follows: pieces of porcine buccal and sublingual mucosa were cut with a size of 2.5 × 2.5 cm and were fixed on two planks, respectively. One plank was fixed on a stainless-steel base, and the other was connected with a firm thread, which fastened a light plastic beaker through a fixed little crown block. A total of 0.3 g of sample were placed between two pieces of tissue and then slightly pressed on an upper plank by hand. Water was then dropped into the beaker with a constant flow of 1.0 mL/min until the two planks were pulled apart due to the gravity of the water on the beaker. The beaker with water was weighed at the end, and the mucoadhesive force (*F*, mN/cm^2^) was calculated by the following Equation (5):

(5) $$F=\frac{W\times g}{A}\;$$

where *W* is the mass of water, *g* is the acceleration due to gravity, and *A* is the surface area of the applied formulation. The mucoadhesive force was presented as mean values ± SD of three replicates for each tissue sample.

#### 4.4.7. Syringeability/Extrudability Study

This test measures the force required for the sample to be removed from the container. The syringeability (E) of the development formulation was assessed by measuring the weight required to remove the hydrogel from a 2 mL syringe. Formulation (500 g) was carefully loaded into syringes avoiding the formation of air bubbles. The device was vertically placed on a support, and known weights (200 and 500 g) were added to their plunges. The syringeability of the formulation was calculated according to the Equation (6)

(6) $$E=\frac{W\;}{A}\;$$

where *W* is the weigh applied (g) to extrude the sample from the syringe and *A* is the area (cm^2^) of the extruded hydrogel from the syringe. The data obtained were presented as the mean ± SD of three replicates.

#### 4.4.8. Rheological Analysis

Rheological characterization of the gel samples (prior to being reticulated, after reticulation, and finally, the gel reticulated and extruded) was carried out in duplicate using a Thermo Scientific Haake Rheostress 1 rheometer (Thermo Fischer Scientific, Kalsruhe, Germany). Steady-state measurements were made with a fixed plate and a mobile upper plate (PP60/2° Ti; 60 mm diameter and 2° angle). The device was controlled by Haake Rheowin^®^ Job Manager v. 4.87 software. The shear stress (*τ*) was measured as a function of the shear rate (γ). Viscosity curves (*η* = f(*γ*)) and flow curves (*τ* = f(*γ*)) were recorded at 25 ± 0.1 °C. The shear rate ramp program included a 1 min ramp-up period from 0 to 100 s^−1^, 1 min constant shear rate period at 100 s^−1^, and 1 min ramp-down from 100 to 0 s^−1^. Representative mathematical models were fitted to flow curves [46]. The best fitting model was selected based on the correlation coefficient (observed vs. predicted) and chi-square value. Equation (7) shows the Cross model. Steady-state viscosity (η, Pa.s) was determined from the constant shear section at 100 s^−1^.

(7) $$\tau =\gamma ˙\cdot \;\left(\;\eta \infty +\eta 0-\eta \infty \right)/\left(1+{\left(\gamma ˙/\gamma_{0}\right)}^{n}\right)$$

where *η* is the dynamic viscosity (Pa·s), *τ* is the shear stress (Pa), *η0* is the zero-shear rate viscosity, *η∞* is the infinity shear rate viscosity *γ*, is the shear rate (1/s).

### 4.5. In Vitro Release Study

Amber glass Franz diffusion cells (FDC 400, Crown Glass, Somerville, NY, USA) with an active diffusion area of 0.64 cm^2^ (*n* = 6) were used for the in vitro release study. Two membranes were evaluated: nylon membrane and polyethersulfone (PES) 0.45 µm pore size. The receptor fluid consisted of a 0.06 M phosphate buffer solution (PBS) (pH 7.4), which was continuously stirred at 500 rpm. The system was kept stable at a temperature of 37 ± 0.5 °C by a thermostatic water-bath. The system was allowed to equilibrate for at least 30 min before applying the hydrogel, and air bubbles entrapped below the membranes were removed. Sink conditions were held throughout the experiments, and 200 mg ± 10 mg of ketorolac formulation was accurately applied to the membranes in the donor compartment. Parafilm was used to avoid evaporation by sealing the donor compartment and the sampling ports. Samples (300 µL) were collected at times 30 min, 1 h and the following, 2, 3, 5, 6, and 22 h, and the same volume was immediately replaced with PBS after the removal of each sample. The KT was determined by a previously validated HPLC method using an isocratic elution of 1:1 (acetonitrile: acidified water) at the flux 1 mL per minute; acetonitrile was supplemented with triethylamine (0.065%), and water was acidified with 0.165% of acetic glacial acid. A Waters 717 plus autosampler equipped with a Waters UV-vis detector 2487 and Waters 515 pumps was used. A YMC-Pack Pro C18 column was used (25 cm, 4.6 mm and 5 μm), the detector was set at 314 nm, and the injection volume was 10 µL [16]. The experimental data were fitted to different mathematical models: hyperbolic, first-order, and Higuchi (Equations (8)–(10), respectively). These models have already been verified for the gel in other available studies [45]. The best-fitting model was chosen according to the correlation coefficient (r^2^) value.

(8) $$Rt=\frac{R\infty \cdot t}{K_{d\;}+\;t}$$

(9) $$Rt=R\infty \cdot \;\left(1-e^{K\times t)}\right)$$

(10) Rt=R∞· Kh· t12

where *R∞* is the maximum amount of drug released, *Rt* is the amount of drug released at time *t*, *K Kd*, and *Kh* are the release rate constant.

### 4.6. Ex Vivo Permeation through Buccal/Sublingual Mucosa

The penetration of ketorolac through the oral mucosa (buccal and sublingual) was evaluated by the infinite dose approach (donor compartment dose 150 mg formulation/cm^2^). The experimental conditions were the same as those indicated in the release study (Section 2.5) with sampling times from 1 to 6 h.

Once the permeation data were available (KT amount per cm^2^ versus time), the permeability coefficient (*Kp*) was calculated with the following equation:

(11) $$K_{p}=\frac{J}{C_{0}\times A}$$

where *Kp* is the permeability coefficient of KT through the membranes, *C_0_* (µg/mL) is the initial concentration of ketorolac in the gel, *A* is the surface of the diffusion area, and *J* (µg/cm^2^/h) is the transmucosal flux.

The steady state plasma concentration (*Css*) was calculated assuming areas of application of 1 cm^2^ and 2 cm^2^ according to the Equation:

(12) $$C_{ss}=\frac{J.TSA}{Clp.\;A}$$

where *J* (µg/h) is the flux, *Css* is the steady state plasma concentration, *TSA* (cm^2^) is the theoretical surface area of application, *Clp* (mL/min) is the human plasma clearance of KT, and *A* (cm^2^) is the diffusion area of the Franz cells.

### 4.7. Evaluation of the Drug Amount Released and Retained in the Buccal/Sublingual Mucosa under an Artificial Saliva Flow

The evaluation of the retained amount of KT in different mucosal membranes was carried out under a constant saliva flow, in order to better mimic the in vivo conditions.

The artificial saliva was prepared by mixing 8.0 g of sodium chloride, 0.19 g of potassium monobasic phosphate, and 2.38 g of dibasic sodium phosphate in 1 L of purified water, and the pH was found to be 6.8 [47]. The experimental procedure was carried out at 37 °C. The mucosa was held on a shovel using forceps and 0.5 g of the hydrogel, corresponding to 5 mg of ketorolac, was applied to a surface of 2.54 cm^2^. Using a peristaltic pump, the saliva was passed to the mucosa area with gel at the flux of 0.24 mL/min [48]. Samples of the saliva that had been in contact with the mucosa and the hydrogel were collected every 10 min for one hour. After 60 min, being in the same conditions as in the in vitro permeation test, the mucosal tissue was taken, the formulation excess was removed, and the tissue surface was cleaned. Finally, the mucosa was extracted with 1mL of mobile phase and analyzed by the HPLC method.

### 4.8. Mucosa Histology Evaluation

The buccal and sublingual mucosa histology was evaluated at two stages. This was once the mucous had been obtained from the pig, in order to evaluate the basal state of the tissue, and the other stage was that at the end of permeation experiment, it was checked that no alterations had taken place throughout the experiment. To conduct the tissue histology, the following procedure was used [49]. The tissues were immersed in 4% buffered paraformaldehyde for 24 h to fix them and then, after dehydration, these tissues were embedded in paraffin and cut at 6µm, stained, and mounted on DPX (Sigma Aldrich, Saint Louis, MI, USA). Samples were observed under the microscope (Olympus BX41 and camera Olympus XC50) on a blind-coded sample.

## Figures and Tables

**Table 1:** Variables and measured responses for the 16 gel experiment formulations. The alginate and the crosslinking agent (CaCl_2_) were mixed in a 1:1 for the final formulation.

Formulation	Alginate [%]	CaCl_2_ [mM]	Gelation [Yes/No]	Physical Stability [Yes/No]	Viscosity	pH
G1	0.5	4	No	-	-	-
G2	0.5	8	No	-	-	-
G3	0.5	10	No	-	-	-
G4	0.5	12	No	-	-	-
G7	1	4	No	-	-	-
G8	1	8	Yes	No	-	-
G9	1	10	Yes	No	-	-
G10	1	12	Yes	No	-	-
G13	2	4	No	-	-	-
G14	2	8	Yes	Yes	Fluid	6.90
G15	2	10	Yes	Yes	Soft	6.99
G16	2	12	Yes	Yes	Thick	7.72

G15: Selected formulation.

**Table 2:** Results of the modelling for the swelling ratio fitting a hyperbola model.

	Swelling Ratio (Mean ± SD)		
Parameters	pH 5.5	pH 7.4	pH 8.0
B_max_ (%)	52.57±	47.17±	17.02±
Kd (min)	1.55±	1.79	
R^2^	0.787	0.972	0.84

B_max_: the maximum swelling ratio; Kd: the time required to obtain half of the swelling ratio.

**Table 3:** Results of the rheological study of the hydrogels before and after reticulation, and the reticulated gel after being extruded: best-fit modelling and viscosity at 25 °C.

Formulation	Rheological Behaviour and Model		Viscosity at 100 s^−1^ [Pa·s]
	Stretch Ramp-Up	Stretch Ramp-Down	
KT G15 gel previous reticulation	Pseudoplastic (Cross, *r* = 1.0000)	Pseudoplastic (Cross, *r* = 1.0000)	0.94 ± 1.5
KT G15 gel	Pseudoplastic (Cross, *r* = 0.9999)	Pseudoplastic (Cross, *r* = 1.0000)	1.88 ± 24.5
KT G15 gel extruded	Pseudoplastic (Cross, *r* = 0.9999)	Pseudoplastic (Cross, *r* = 1.0000)	1.68 ± 13.9

**Table 4:** The correlation coefficient (r^2^) value in the different kinetic methods studied.

Kinetic Models	Nylon
Hyperbolic	0.9721
First-order	0.9973
Higuchi	0.897

**Table 5:** Drug release parameters estimated from a first-order kinetics for the nylon membrane.

Parameters	Values ± SD
Y_max_ (µg/cm^2^)	2666 ± 25
K (h^−1^)	0.225 ± 0.027
Half Life (h)	3.08
R^2^	0.997

Drug release parameters from KT hydrogel according to a first-order kinetics: K = release rate constant, Y_max_ = total amount of drug released. Values represent means ± standard deviation (*n* = 6).

**Table 6:** Permeation parameters of the KT hydrogel and statistical analysis of non-parametric *t*-test between buccal and sublingual mucosae.

		Buccal			Sublingual			
Parameters	Units	Median	Min	Max	Median	Min	Max	*p*-Value
J	µg/h	92.89	82.35	100.67	264.8	214.62	316.09	*p* < 0.0001
J/SUP	µg/h/cm^2^	145.14	128.67	157.3	413.75	335.34	493.89	*p* < 0.0001
Kp	cm/h	0.0143	0.0129	0.0157	0.0414	0.0335	0.0494	*p* < 0.0001
Q6	µg/cm^2^	547.32	534.73	637.8	1863.14	1652.41	2354.12	*p* < 0.0001
Css 1 cm^2^	µg/mL	0.078	0.07	0.085	0.225	0.182	0.268	*p* < 0.0001
Css 2 cm^2^	µg/mL	0.155	0.14	0.171	0.45	0.365	0.537	*p* < 0.0001

where J (µg/h) is the flux (SUP = diffusional area), Css is the steady state plasma concentration (Css), Q6 is the amount of KT permeated at 6 h, and Kp is the permeability coefficient of ketorolac through the membranes.

**Table 7:** Final composition of the candidate gels. All formulation contained KT (ketorolac) at final concentration of 1% *w*/*w*.

Formulation	G1	G2	G3	G4	G5	G6	G7	G8	G9	G10	G11	G12	G13	G14	G15	G16	G17	G18
Alginate [%]	0.5	0.5	0.5	0.5	0.5	0.5	1	1	1	1	1	1	2	2	2	2	2	2
CaCl_2_ [mM]	4	8	10	12	25	50	4	8	10	12	25	50	4	8	10	12	25	50

## Data Availability

The data presented in this study are available on request from the corresponding author. The data are not publicly available due to their being used as part of a doctoral thesis, and they will be available once the thesis has been published.

## References

[1] Torres Lagares D., Barranco Piedra S., Serrera Figallo M.Á., Hita Iglesias P., Martínez-Sahuquillo Márquez A., Gutiérrez Pérez J.L. (2006). Sialolitiasis Parotídea Del Conducto de Stensen. Med. Oral Patol. Oral Y Cirugía Bucal.

[2] Hupp J., Tucker M., Ellis E., Peterson L.J., Ellis E., Hupp J.R., Tucker M.R. (2018). Contemporary Oral and Maxillofacial Surgery.

[3] Thong H.K., Mohamad Mahbob H., Sabir Husin Athar P.P., Tengku Kamalden T.M.I. (2020). Recurrent Submandibular Sialolithiasis in a Child. Cureus.

[4] Orlian A.I., Schaefer M., Golub J. (1998). Multiple Bilateral Sialoliths of the Submandibular Ducts. N. Y. State Dent. J..

[5] Koch M., Mantsopoulos K., Müller S., Sievert M., Iro H. (2022). Treatment of Sialolithiasis: What Has Changed? An Update of the Treatment Algorithms and a Review of the Literature. J. Clin. Med..

[6] Alvarez Echazú M.I., Olivetti C.E., Anesini C., Perez C.J., Alvarez G.S., Desimone M.F. (2017). Development and Evaluation of Thymol-Chitosan Hydrogels with Antimicrobial-Antioxidant Activity for Oral Local Delivery. Mater. Sci. Eng. C Mater. Biol. Appl..

[7] Parhi R. (2017). Cross-Linked Hydrogel for Pharmaceutical Applications: A Review. Adv. Pharm. Bull..

[8] Lu L., Yuan S., Wang J., Shen Y., Deng S., Xie L., Yang Q. (2018). The Formation Mechanism of Hydrogels. Curr. Stem Cell Res. Ther..

[9] Goh C.H., Heng P.W.S., Chan L.W. (2012). Alginates as a Useful Natural Polymer for Microencapsulation and Therapeutic Applications. Carbohydr. Polym..

[10] Chaturvedi K., Ganguly K., More U.A., Reddy K.R., Dugge T., Naik B., Taminabhavi T.M., Noolvi M.N., Hasnain M.S., Nayak A.K. (2019). Sodium Alginate in Drug Delivery and Biomedical Areas. Natural Polysaccharides in Drug Delivery and Biomedical Application.

[11] Park K., Robinson J.R. (1984). Bioadhesive Polymers as Platforms for Oral-Controlled Drug Delivery: Method to Study Bioadhesion. Int. J. Pharm..

[12] Chickering D.E., Mathiowitz E. (1995). Bioadhesive Microspheres: I. A Novel Electrobalance-Based Method to Study Adhesive Interactions between Individual Microspheres and Intestinal Mucosa. J. Control. Release.

[13] Hasnain M.S., Jameel E., Mohanta B., Dhara A.K., Alkahtani S., Nayak A.K. (2020). Alginates: Sources, Structure, and Properties. Alginates in Drug Delivery.

[14] Trindade P.A.K., Giglio F.P.M., Colombini-Ishikiriama B.L., Calvo A.M., Modena K.C.S., Ribeiro D.A., Dionísio T.J., Brozoski D.T., Lauris J.R.P., Faria F.A.C. (2012). Sublingual Ketorolac and Sublingual Piroxicam Are Equally Effective for Postoperative Pain, Trismus, and Swelling Management in Lower Third Molar Removal. Oral Surg. Oral Med. Oral Pathol. Oral Radiol..

[15] Penniston S.G., Hargreaves K.M. (1996). Evaluation of Periapical Injection of Ketorolac for Management of Endodontic Pain. J. Endod..

[16] Rogers M.J., Johnson B.R., Remeikis N.A., Begole E.A. (1999). Comparison of Effect of Intracanal Use of Ketorolac Tromethamine and Dexamethasone with Oral Ibuprofen on Post Treatment Endodontic Pain. J. Endod..

[17] National Center for Biotechnology Information PubChem Compound Summary for CID 84003, Ketorolac Tromethamine. https://pubchem.ncbi.nlm.nih.gov/compound/Ketorolac-tromethamine.

[18] National Center for Biotechnology Information PubChem Compound Summary for CID 133126842 Sodium Alginate. https://pubchem.ncbi.nlm.nih.gov/compound/133126842.

[19] Ghannam M.G., Singh P. (2022). Anatomy, Head and Neck, Salivary Glands.

[20] LLS Health Technical Team How Sensory Properties Are Driving Topical Drug Product Development. https://www.lubrizol.com/Health/Blog/2020/12/How-Sensory-Properties-are-Driving-Topical-Drug-Product-Development.

[21] Li J., Suo Z., Vlassak J.J. (2014). Stiff, Strong, and Tough Hydrogels with Good Chemical Stability. J. Mater. Chem. B.

[22] Baus R.A., Zahir-Jouzdani F., Dünnhaupt S., Atyabi F., Bernkop-Schnürch A. (2019). Mucoadhesive Hydrogels for Buccal Drug Delivery: In Vitro-In Vivo Correlation Study. Eur. J. Pharm. Biopharm..

[23] El Moussaoui S., Fernández-Campos F., Alonso C., Limón D., Halbaut L., Garduño-Ramirez M.L., Calpena A.C., Mallandrich M. (2021). Topical Mucoadhesive Alginate-Based Hydrogel Loading Ketorolac for Pain Management after Pharmacotherapy, Ablation, or Surgical Removal in Condyloma Acuminata. Gels.

[24] Foudazi R., Zowada R., Manas-Zloczower I., Feke D.L. (2023). Porous Hydrogels: Present Challenges and Future Opportunities. Langmuir.

[25] Roberge C.L., Kingsley D.M., Cornely L.R., Spain C.J., Fortin A.G., Corr D.T. (2022). Viscoelastic Properties of Bioprinted Alginate Microbeads Compared to Their Bulk Hydrogel Analogs. J. Biomech. Eng..

[26] Nindiyasari F., Fernández-Díaz L., Griesshaber E., Astilleros J.M., Sánchez-Pastor N., Schmahl W.W. (2014). Influence of Gelatin Hydrogel Porosity on the Crystallization of CaCO_3_. Cryst. Growth Des..

[27] Choi N.W., Kim J., Chapin S.C., Duong T., Donohue E., Pandey P., Broom W., Hill W.A., Doyle P.S. (2012). Multiplexed Detection of MRNA Using Porosity-Tuned Hydrogel Microparticles. Anal. Chem..

[28] Da Silva M.A., Bierhalz A.C.K., Kieckbusch T.G. (2009). Alginate and Pectin Composite Films Crosslinked with Ca^2+^ Ions: Effect of the Plasticizer Concentration. Carbohydr. Polym..

[29] El Moussaoui S., Mallandrich M., Garrós N., Calpena A.C., Lagunas M.J.R., Fernández-Campos F. (2021). Hpv Lesions and Other Issues in the Oral Cavity Treatment and Removal without Pain. Int. J. Mol. Sci..

[30] Patel V.F., Liu F., Brown M.B. (2011). Advances in Oral Transmucosal Drug Delivery. J. Control. Release.

[31] Moles-Aranda C., Calpena-Campmany A., Halbaut-Bellowa L., Díaz-Tomé V., Otero-Espinar F., Morales-Molina J., Clares-Naveros B. (2020). Novel Polymeric Formulation for Removal of Gastrointestinal Polyps by Digestive Endoscopy. Pharmaceutics.

[32] Mallandrich M., Fernández-Campos F., Clares B., Halbaut L., Alonso C., Coderch L., Garduño-Ramírez M.L., Andrade B., del Pozo A., Lane M.E. (2017). Developing Transdermal Applications of Ketorolac Tromethamine Entrapped in Stimuli Sensitive Block Copolymer Hydrogels. Pharm. Res..

[33] O’Donovan S., Ferrara A., Larach S., Williamson P. (1994). Intraoperative Use of Toradol® Facilitates Outpatient Hemorrhoidectomy. Dis. Colon Rectum.

[34] Limón D., Jiménez-Newman C., Calpena A.C., González-Campo A., Amabilino D.B., Pérez-García L. (2017). Microscale Coiling in Bis-Imidazolium Supramolecular Hydrogel Fibres Induced by the Release of a Cationic Serine Protease Inhibitor. Chem. Commun..

[35] Lau W.M., Ng K.W., Dragicevic N., Maibach H.I. (2017). Finite and Infinite Dosing. Percutaneous Penetration Enhancers Drug Penetration into/through the Skin.

[36] PubChem Database Ketorolac|C15H13NO3—PubChem. https://Pubchem.Ncbi.Nlm.Nih.Gov/Compound/3826.

[37] PubChem Database Ketorolac Tromethamine|C19H24N2O6—PubChem. https://Pubchem.Ncbi.Nlm.Nih.Gov/Compound/Ketorolac-Tromethamine.

[38] (2008). TORADOL (Ketorolac Tromethamine Tablets).

[39] Harris D., Robinson J.R. (1992). Drug Delivery via the Mucous Membranes of the Oral Cavity. J. Pharm. Sci..

[40] Cordero J.A., Alarcon L., Escribano E., Obach R., Domenech J. (1997). A Comparative Study of the Transdermal Penetration of a Series of Nonsteroidal Antiinflammatory Drugs. J. Pharm. Sci..

[41] Miranda M., Cardoso C., Vitorino C. (2020). Quality and Equivalence of Topical Products: A Critical Appraisal. Eur. J. Pharm. Sci..

[42] Li W.-Z., Zhao N., Zhou Y.-Q., Yang L.-B., Wang X.-N., Hao B.-H., Peng K., Zhang C.-F. (2012). Post-Expansile Hydrogel Foam Aerosol of PG-Liposomes: A Novel Delivery System for Vaginal Drug Delivery Applications. Eur. J. Pharm. Sci..

[43] Mei L., Chen J., Yu S., Huang Y., Xie Y., Wang H., Pan X., Wu C. (2017). Expansible Thermal Gelling Foam Aerosol for Vaginal Drug Delivery. Drug Deliv..

[44] Yang T.-T., Cheng Y.-Z., Qin M., Wang Y.-H., Yu H.-L., Wang A.-L., Zhang W.-F. (2017). Thermosensitive Chitosan Hydrogels Containing Polymeric Microspheres for Vaginal Drug Delivery. Biomed. Res. Int..

[45] Costa P., Sousa Lobo J.M. (2001). Modeling and Comparison of Dissolution Profiles. Eur. J. Pharm. Sci..

[46] Park E.K., Song K.W. (2010). Rheological Evaluation of Petroleum Jelly as a Base Material in Ointment and Cream Formulations: Steady Shear Flow Behavior. Arch. Pharm. Res..

[47] Marques M.R.C., Loebenberg R., Almukainzi M. (2011). Simulated Biological Fluids with Possible Application in Dissolution Testing. Dissolut. Technol..

[48] Humphrey S.P., Williamson R.T. (2001). A Review of Saliva: Normal Composition, Flow, and Function. J. Prosthet. Dent..

[49] Gómez-Segura L., Parra A., Calpena A.C., Gimeno Á., Boix-Montañes A. (2020). Carprofen Permeation Test through Porcine Ex Vivo Mucous Membranes and Ophthalmic Tissues for Tolerability Assessments: Validation and Histological Study. Vet. Sci..

